# Optimal regulation of bipedal walking speed despite an unexpected bump in the road

**DOI:** 10.1371/journal.pone.0204205

**Published:** 2018-09-26

**Authors:** Osman Darici, Hakan Temeltas, Arthur D. Kuo

**Affiliations:** 1 University of Calgary Faculty of Kinesiology, Calgary, Alberta, Canada; 2 Istanbul Technical University, Faculty of Electrical & Electronics Eng., Istanbul, Turkey; Purdue University, UNITED STATES

## Abstract

Bipedal locomotion may occur over imperfect surfaces with bumps or other features that disrupt steady gait. An unexpected bump in the road is generally expected to slow down most types of locomotion. On wheels, speed may be regained quite readily with “cruise control” performed in continuous time. But legged locomotion is less straightforward, because the stance leg may be under-actuated, and the continuous-time dynamics are periodically disrupted by discrete ground contact events. Those events may also afford good control opportunities, albeit subject to the delay between discrete opportunities. The regulation of walking speed should ideally use these opportunities to compensate for lost time, and with good economy if possible. However, the appropriate control strategy is unknown. Here we present how to restore speed and make up for time lost going over a bump in the road, through discrete, once-per-step control. We use a simple dynamic walking model to determine the optimal sequence of control actions—pushing off from the leg at the end of each stance phase—for fast response or best economy. A two-step, deadbeat sequence is the fastest possible response, and reasonably economical. Slower responses over more steps are more economical overall, but a bigger difference is that they demand considerably less peak power. A simple, reactive control strategy can thus compensate for an unexpected bump, with explicit trade-offs in time and work. Control of legged locomotion is not as straightforward as with wheels, but discrete control actions also allow for effective and economical reactions to imperfect terrain.

## Introduction

Humans and other bipeds may occasionally encounter bumps or other surface imperfections that slow down steady gait. A bump in the road might be avoided if seen in advance, but otherwise requires reactive control. Assuming balance is maintained, control is needed to regulate walking speed despite disturbances. This is not as simple as automotive cruise control, where continuous feedback control can be applied to the driven wheels. With legged locomotion, substantive control may have to wait until the next discrete footfall, and the cumulative delay from multiple bumps may substantially reduce the average walking speed. A bump’s effect on walking, and the appropriate way to regain the intended average speed, are considerably different with legs. Examination of these dynamics might provide insight on how humans walk, and what control strategies robots might reasonably employ after a bump in the road.

Legged locomotion often entails pendulum-like dynamics [1]. In humans [2] and some robots [3–7], the stance leg behaves much like an underactuated, inverted pendulum. The transition between steps entails a dissipative collision between swing leg with ground, and energy may be restored by performing positive work during the single stance phase [3]. Dynamic stability is largely a matter of placing the swing leg for the next footfall, such as by actively swinging it forward in proper amount [8], or by enforcing a virtual holonomic constraint relative to the stance leg (“hybrid zero dynamics,” [4]). Virtual constraints result in relatively fixed step lengths, not unlike a “rimless wheel” [1], and with high robustness against falling due to bumps [9]. But even if falls are avoided as such, bumps will generally still disrupt forward speed. To focus on speed regulation, the present study will be limited to dynamic walking with an inverted pendulum supporting the COM, moving forward in the sagittal plane, and with fixed step lengths for guaranteed stability.

If it is not anticipated, a discrete bump can disrupt walking speed in several ways. First, the vertical height means slower speed atop the bump, because the bump’s greater potential energy comes at the expense of the inverted pendulum’s kinetic energy. Second, the leg landing on the bump would be expected to sweep through a larger angular arc and take further time. A third effect is that an unexpected bump’s height may cause footfall onto the bump to occur earlier than expected. On level ground, humans normally begin pushing off with the trailing leg before the leading leg’s heelstrike [10,11]. This is economical because the pre-emptive push-off actually reduces the energy dissipated by collision [12]. An early collision would be less economical, even if the push-off energy were stored elastically [13] and released late, immediately after collision [14]. An additional effect is less speed gained from the previous step’s stance phase, due to the abbreviated fall of that leg’s inverted pendulum. The overall result is a considerable loss in walking speed while going over the bump.

There may be several ways to make up for a loss of speed. If there is no time constraint, walking speed will asymptotically return to normal without any compensation, due to the repeated effect of nominal push-offs [15]. But some tasks do involve a time or speed constraint, for example if a robot is commanded to reach its target at designated time, or a person wishes to walk with a companion and match their speed. In such cases, each bump and delayed speed response would cause additional time loss. This calls for a control strategy that can both restore speed and make up for lost time, perhaps with appropriate objectives for response time or economy. The purpose of the present study was therefore to use dynamic optimization to examine the possible strategies to react to an unanticipated bump in the road. Here we present a simple dynamic walking model, and quantitatively demonstrate the loss of speed and time caused by the bump. We then describe the optimal response strategies, in terms of discrete control sequences, to restore speed and make up for lost time. The result is a set of relatively simple, reactive control sequences that maintain a consistent average speed despite unanticipated imperfections in the walking surface.

## Materials and methods

**Fig 1:**
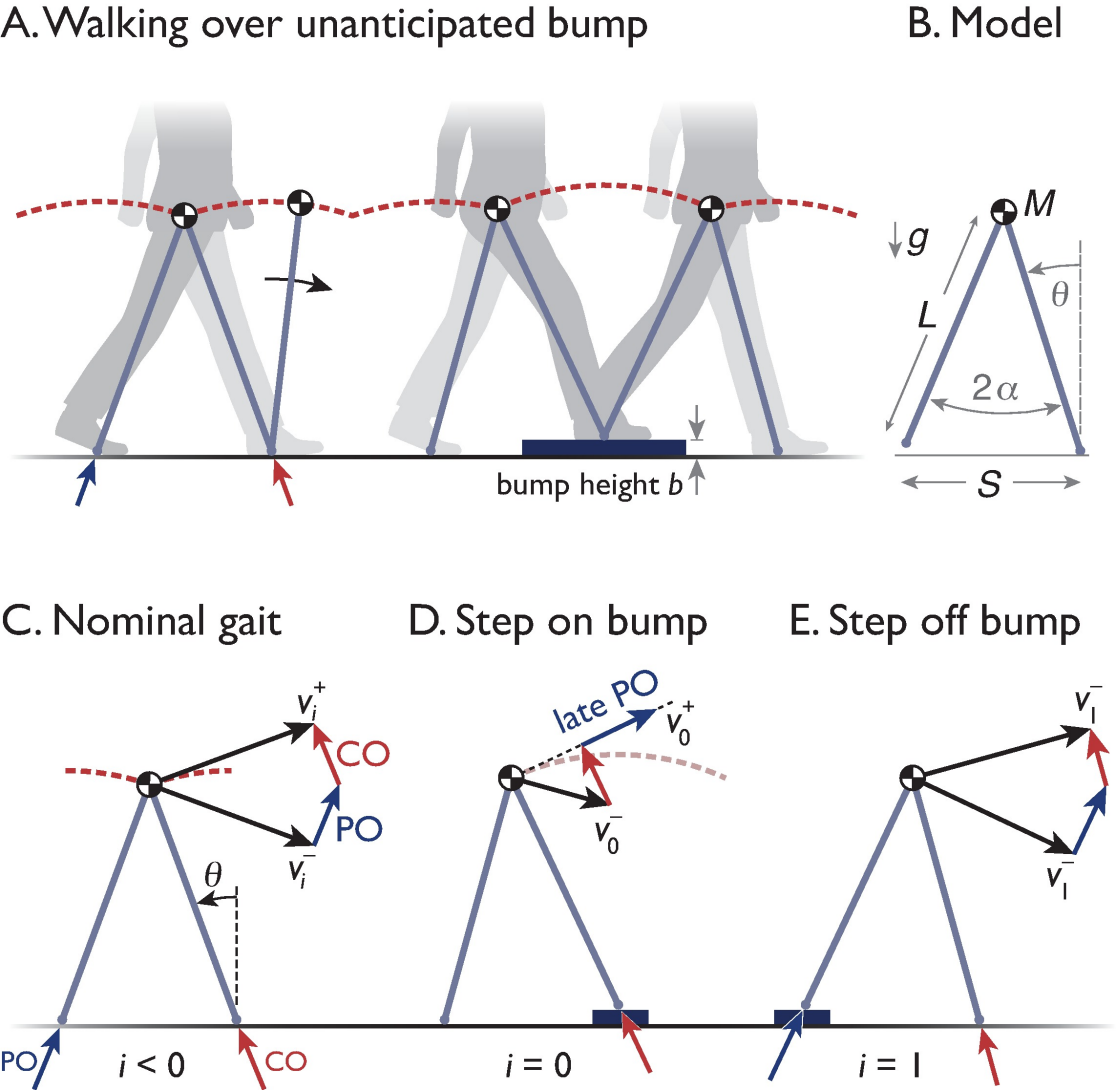
Model of walking on a bump. (A.) The “simplest walking model” treats the stance leg as an inverted pendulum, so that the body center of mass (COM) moves in an arc each step. (B.) Stance leg angle is measured with angle *θ*, with fixed angle 2*α* between the legs at double support, yielding fixed step length *S*. Point mass *M*, leg length *L*, and gravitational acceleration *g* serve as base units for non-dimensionalization of model equations. (C.) Nominal gait has a step-to-step transition between pendulum-like arcs. The COM velocity is redirected from forward-and-downward $v_{i}^{-}$, to forward-and-upward $v_{i}^{+}$, through active, impulsive trailing leg push-off (PO), followed by impulsive leading leg collision (CO). (D.) Bump occurs on step *i* = 0, with an early collision, so that push-off energy is released late, resulting in post-bump velocity $v_{0}^{+}$. (E.) Stepping off the bump occurs with pre-emptive push-off. Optimal push-off sequence is computed to minimize total push-off work.

We applied dynamic optimization to predict how to recover from a bump. The dynamics of the task were governed by a walking model with an inverted pendulum for a stance leg (Fig 1A), nominally powered by pushing from the leg with each step, but with steady gait disturbed by an unexpected bump. The optimization objective was to recover from the bump by modulating a sequence of push-off control actions, for either fast response or for economy.

### Model dynamics

The model is based on a simple inverted pendulum (Fig 1B). Each step begins with a discrete step-to-step transition followed by a continuous-time, pendulum-like stance phase. The stance phase is governed by non-dimensionalized, linearized, inverted pendulum dynamics

$$\ddot{\theta }-\theta =0$$ (1)

where *θ* is the stance leg angle, measured counterclockwise with respect to vertical, with body mass *M*, gravitational acceleration *g*, and leg length *L* serving as base units. Thus, time *t* may be regarded as having units *g*^−0.5^*L*^0.5^. On level ground, a stance phase has initial angle *α* and final angle −*α*, thus fixing the inter-leg angle and step length (2*α* and *S* = 2 sin *α*, respectively). The stance leg behaves similar to the “simplest walking model” [15], with a swing leg of infinitesimal mass, except constrained here to yield a fixed step length, like the rimless wheel model [1]. The angular speed of the pendulum determines the COM velocity’s magnitude, $v=-\dot{\theta }$ (forward motion corresponds to clockwise/negative angular velocity).

The step-to-step transition terminates the preceding step’s stance phase and initiates the next one. The transition consists of two impulsive events that occur in quick succession. In most cases, the step-to-step transition consists of a pre-emptive push-off followed by a heel-strike collision (Fig 1C). The pre-emptive push-off is performed by and along the previous stance leg just before collision. The collision is modeled as a perfectly inelastic collision with ground, which reduces the COM velocity. It is most economical to push off pre-emptively, because it reduces the subsequent collision [15]. This push-off and collision sequence is therefore applied to all steps save one. The exception is stepping on an unanticipated bump (Fig 1D), where the collision occurs earlier than expected and disrupts the pre-emptive push-off. In this case, the push-off occurs late, and impulsively adds speed just after the collision, so that the step-to-step transition consists of a collision and then a late push-off. A full walking step therefore usually starts with a pre-emptive push-off and collision (Fig 1C and 1E), followed by an inverted pendulum phase that ends just before the next leg contacts ground. But when stepping onto the bump, the collision precedes late push-off and the inverted pendulum phase.

The events of a step may be described mathematically in terms of COM velocity. Step number *i* begins with COM velocity $v_{i}^{-}$, which is directed forward and downward, in amount and angle determined by the preceding step’s stance phase. Pre-emptive push-off *u*_*i*_ then acts impulsively along the trailing leg to redirect the COM velocity and yield an intermediate velocity $v_{i}^{0}$,

$$v_{i}^{0}=\sqrt{{\left(v_{i}^{-}\right)}^{2}+2u_{i}}$$ (2)

where *u*_*i*_ is defined as the (mass-normalized) push-off work. Because push-off direction is perpendicular to $v_{i}^{-}$, the associated velocity change is $\sqrt{2u_{i}}$ [15].

This is followed immediately by the collision between leading leg and ground. With the system’s mass concentrated at the COM, the impulse must act along the leading leg, to yield a post-collision velocity $v_{i}^{+}$. For all steps other than onto the bump,

$$v_{i}^{+}=v_{i}^{-}\;\cos\;2\alpha +\sqrt{{2u}_{i}}\;\sin\;2\alpha \;for\;i\neq 0.$$ (3)

For nominal walking on the level, the COM velocities before and after the step-to-step transition are equal to each other, denoted nominal $V=v_{i}^{-}=v_{i}^{+}$, and the nominal pre-emptive push-off work is

$$U=\frac{1}{2}V^{2}\;\tan^{2}\alpha .$$ (4)

An unexpected bump disrupts the model’s normal energy input (Fig 1D). The bump causes the heelstrike collision to occur earlier than normal, preventing pre-emptive push-off. Therefore, the leading leg collision first redirects COM velocity along a new pendular arc. The trailing leg push-off energy may then be released late, in amount $u_{0}^{+}$ (with the plus sign indicating the post-collision timing) impulsively increasing speed along that new arc:

$$v_{0}^{+}=\sqrt{{\left(v_{0}^{-}\;\cos\;2\alpha \right)}^{2}+{2u}_{0}^{+}}\;for\;i=0.$$ (5)

We treat $u_{0}^{+}$ as defaulting to amount *U*, as if it were released from nominally stored elastic energy. This is because humans store much of the push-off energy in the Achilles tendon during much of the stance phase [13,14], so that the same amount of energy might still be released, despite the unanticipated collision atop the bump. And when there is a controlled compensation, we treat $u_{0}^{+}$ as a selectable quantity included in the control strategy. In that case, late push-off might be modulated or augmented by quickly exerting hip torque between trunk and stance leg [1]. And after the bump, all subsequent steps have pre-emptive push-off. These subsequent push-offs are also treated as controllable, as if once the unexpected bump has occurred, attention is heightened, allowing an optimal recovery to be executed (Fig 1E).

Each step-to-step transition determines the speed and timing of the next stance phase, which is entirely ballistic and pendulum-like. The COM velocity $v_{i}^{+}$ (Eqs 3 or 5), serves as the initial speed for the continuous-time, inverted pendulum phase. The initial stance leg angle depends on bump height *b* and the fixed step length *S* (as does the final angle at end of stance). The initial stance angle for step *i* is perturbed by an angle *δ*_*i*_,

$$\delta_{0}=\sin^{-1}\frac{b}{S},\;\delta_{i}=0\;for\;i\neq 0.$$ (6)

The step time *τ*_*i*_ is defined as the time for the inverted pendulum to move between the initial and final angles. Using the linearized inverted pendulum dynamics,

$$\tau_{i}=\log\frac{\alpha -\delta_{i+1}+\sqrt{{\left(v_{i}^{+}\right)}^{2}-2\alpha {(\delta_{i}+\delta }_{i+1})+\delta_{i+1}^{2}-\delta_{i}^{2}}}{v_{i}^{+}-\alpha -\delta_{i}}$$ (7)

Solving the equation of motion with this step time, the velocity at end of stance, or equivalently the beginning of the next step-to-step transition, is

$$v_{i+1}^{-}=\frac{1}{2}\left(e^{-\tau_{i}}(v_{i}^{+}-\alpha -\delta_{i})+e^{\tau_{i}}(v_{i}^{+}+\alpha +\delta_{i})\right).$$ (8)

The average speed for a step *i* is then the fixed step length *s* divided by step time *τ*_*i*_, and the average speed for *N* steps is *N* ∙ *s* divided by the total time of those steps, ∑*τ*_*i*_.

The preceding dynamics show how an unanticipated bump substantially affects gait. If the late push-off is not increased from nominal ($u_{0}^{+}=U$), the next step will be of substantially reduced speed $v_{0}^{+}<V$ (compare Eqs 3 and 5) and have slower step time *τ*_0_. More push-off is needed if nominal speed is to be re-gained. This is illustrated by the limiting case of a bump approaching zero height, but still causing early collision. Applying $v_{0}^{-}=V$ to Eq (5),

$$u_{0}^{+}=\frac{1}{2}V^{2}\;\sin^{2}\;2\alpha ,$$ (9)

which is four times greater than nominal (Eq 4, assuming a small angle approximation for *α*). This reiterates the considerable advantage of pushing off pre-emptively, as described previously [15].

We defined a nominal, steady walking gait on level ground. The nominal parameters are denoted *u*_*i*_ = *U*, *τ*_*i*_ = *T*, $v_{i}^{-}=v_{i}^{+}=V$, etc. To approximate typical human walking at 1.5 m/s with leg length *L* of 1 m, step length 0.79 m, and step time 0.53 s, we selected angle *α* = 0.41, *U* = 0.0342 *MgL*, *T* = 1.66 *g*^−0.5^*L*^0.5^, *V* = 0.601 *g*^0.5^*L*^0.5^ where *g* refers to gravitational acceleration. The model steps onto a bump with standard height *B* of 0.025*L* (equivalent to 2.5 cm), which is intended to be small enough that humans might not anticipate it, yet large enough to meaningfully affect gait speed. (Bumps both taller and shorter than *B* are also examined as parameter variations.) Most of the analysis below is based on linearized dynamics for this system, the accuracy of which is also tested below with an optimization performed with fully nonlinear dynamics. The equations were non-dimensionalized using body mass *M*, leg length *L*, and gravitational acceleration *g* as base units, but for convenience, parameter values are reported along with re-dimensionalizing units.

### Optimization problem

Walking over a bump was considered with and without an optimal response. In the No Compensation case, no optimization was performed. All push-offs were therefore applied at nominal amount *U*, with the special case of stepping onto the bump, where the energy was released late, $u_{0}^{+}=U$. In optimal cases, dynamic optimization was used to determine a push-off sequence to walk over the unexpected bump with minimal positive work. The control variables were therefore impulsive push-off work *u*_*i*_ (except for late push-off atop the bump). The constraints were that the final walking speed and total time must match that for nominal level, steady walking, over a designated number of steps of fixed step length.

The number of steps was described by two free parameters, one a delay of *d* steps before the model reacts to the bump, and the second the number *N* of control inputs that form the control policy. Therefore, the control policy *π* consists of *N* work impulses *u*_*i*_, one for each step *i* (with step 0 having late push-off), starting after the delay of *d* steps after the bump. All other push-offs are of nominal energy *U*, even if late. The speed constraints are such that the initial and final conditions (*i* < 0 and *i* ≥ *N* + *d*, respectively) are equal to the nominal, steady speed *V*. The time limit constraint makes up for lost time, so that the total time taken over the *N* + *d* steps, stepping onto and recovering from the bump, is equal to the nominal time to walk the same number of steps on level ground. By the end of the control sequence, the model must walk at the same speed as nominal, and must have caught up with the nominal model on level ground. Each step was also constrained by the walking dynamics.

The optimization formulation is therefore as follows:

$$\pi^{*}=\arg\;\min_{\pi }\sum_{i=d}^{N+d-1}u_{i}$$ (10)

subjectto:

$$\mathrm{Speed}:\;v_{0}^{-}=V,\;v_{N+d-1}^{+}=V$$ (11)

$$\mathrm{Time}\;\sum_{i=0}^{N+d-1}\tau_{i}=T\cdot (N+d)$$ (12)

Dynamics: Eqs (1–7)

The delay parameter is included to test whether there is advantage to compensating immediately to the unexpected bump. The model responds with a delay of *d* steps, where *d* = 0 denotes immediate reaction by modulating (usually increasing) the late push-off just after stepping onto the bump. With any greater delay *d* > 0, late push-off work is not modulated, and is instead set equal to the nominal, level walking case. After the bump, the model is presumed to react with pre-emptive push-offs *u*_*i*_, either in controlled amount if the delay has passed (*i* ≥ *d*), otherwise in nominal amount (*i* < *d*).

The No Compensation case is used as a baseline for comparisons. In this case, only the nominal, non-reactive control is applied. In other words, push-off work always remains at the nominal amount *U*, including the late push-off on the bump ($u_{0}^{+}=U$). The overall work performed is therefore the same as for level ground, but with a considerable loss of speed and time due to the bump. Going over the basic bump *B* (0.025*L* in height), the stance time *τ*_0_ is approximately doubled (factor of 2.003) over nominal step time *T*. And stepping off the bump, the speed *v*_1_ is reduced by 10.46% relative to nominal. Once steady state has been reached (9 steps, error <1%), the model has cumulatively lost time of amount 2.53 *g*^−0.5^*L*^0.5^, so that it is 1.51 steps behind the nominal model walking on level ground. The recovery toward steady state may be explained by the eigenvalue of the step-to-step dynamics (cos 2*α* in Eq 3), 0.682, which describes the factor by which a perturbation is reduced by each step. According to this eigenvalue, it takes 6 steps (or 10.96 dimensionless time) for a perturbation to decay to 10% of its initial value.

## Results

The results of the optimization may be summarized with a few simple rules.

### 1. Recover in a minimum of two steps

**Fig 2:**
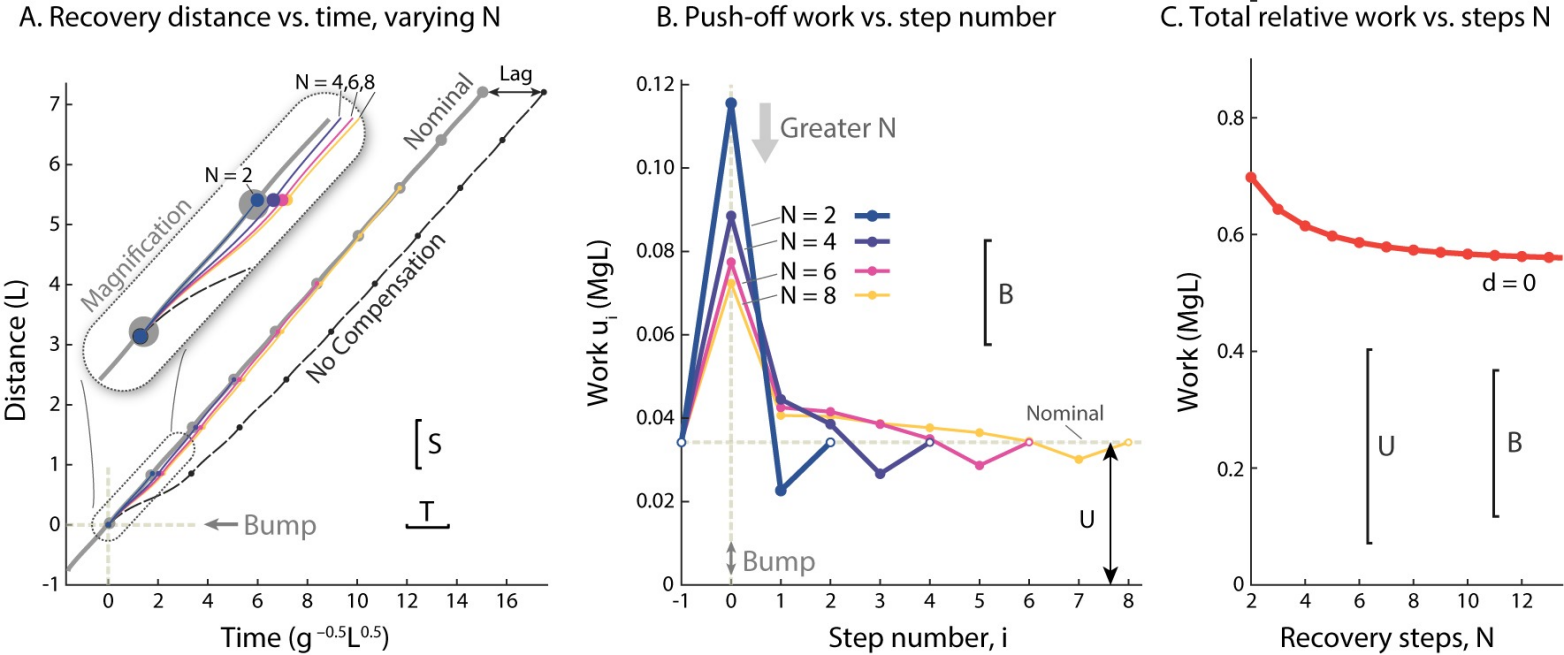
Recovery strategies from a bump in *N* controlled steps. (A.) Cumulative distance vs. time for walking over a basic bump (*B* = 0.025*L*) and recovering within *N* modulated steps (with no delay), with stepping onto the bump occurring at time 0. All gaits except nominal experience an early collision with the bump. The “No Compensation” strategy applies nominal work *U* for all steps (including mid-stance input at step 0), and eventually regains nominal walking speed, but with a time lag (“Lag”) relative to level walking. (B.) Optimal push-off sequences *u*_*i*_ vs. step number for varying *N*, with the bump occurring at step number 0. (C.) Total relative positive work needed to recover in *N* steps, equivalent to the cumulative work of (B.) but subtracting the nominal work for the same number of steps on the level, *N* ⋅ *U*.

It takes a minimum of two steps to meet the speed and time constraints (Fig 2A). The constraints represent two final conditions, and therefore require at least two degrees of freedom in the control policy. With *N* = 2 and zero delay, the late push-off ($u_{0}^{+}$) onto the basic bump *B* (0.025*L*) requires more than three times as much work (3.372*U*) as nominal, and stepping off the bump requires slightly less (33.7%) than nominal work. The strong push-off is helpful because it helps make up for the considerable time otherwise lost atop the bump. But that push-off alone cannot also restore speed. But with the second (*N*th) push-off, the gait speed is restored to nominal. This constitutes a deadbeat control strategy.

### 2. Start pushing off strong, but end weak

With any number of steps, the optimal strategy is still to start with a strong (if late) push-off, and then to end with a relatively weak push-off (Fig 2B). Again, a strong initial push-off is very helpful for increasing speed and reducing the lost time. The succeeding push-offs are not nearly as strong, and consistently decrease in work magnitude with time, although mostly remaining above normal. Strong push-offs help to regain time, which is regained after *N* − 1 push-offs.

The exception is that the final (*N*th) push-off should be weaker than normal. Having briefly sped up, the model must slow now down to match nominal speed. The final push-off is thus modulated such that the final collision achieves this match. These strategies are *N*-step deadbeat controls.

### 3. Taking more time reduces peak push-off demands

An advantage of taking more steps to recover is a reduction in peak push-off work. As *N* increases, the push-off magnitude decreases considerably (Fig 2B). A longer time or step limit *N* provides additional opportunities between the initial and final push-offs, and these are used to distribute work rather than concentrating it in the initial strong push-off. Thus, if actuator saturation is a concern, it helps to recover over more steps.

However, in terms of total work, taking more steps offers a relatively modest advantage (Fig 2C). Measuring total work relative to the nominal work to take the same number of steps on level ground, the two-step strategy only costs 24.9% more work than 15-step control. Thus, the advantage of taking more steps is far more from reducing peak push-off rather than overall work.

### 4. Delayed recovery is very costly

**Fig 3:**
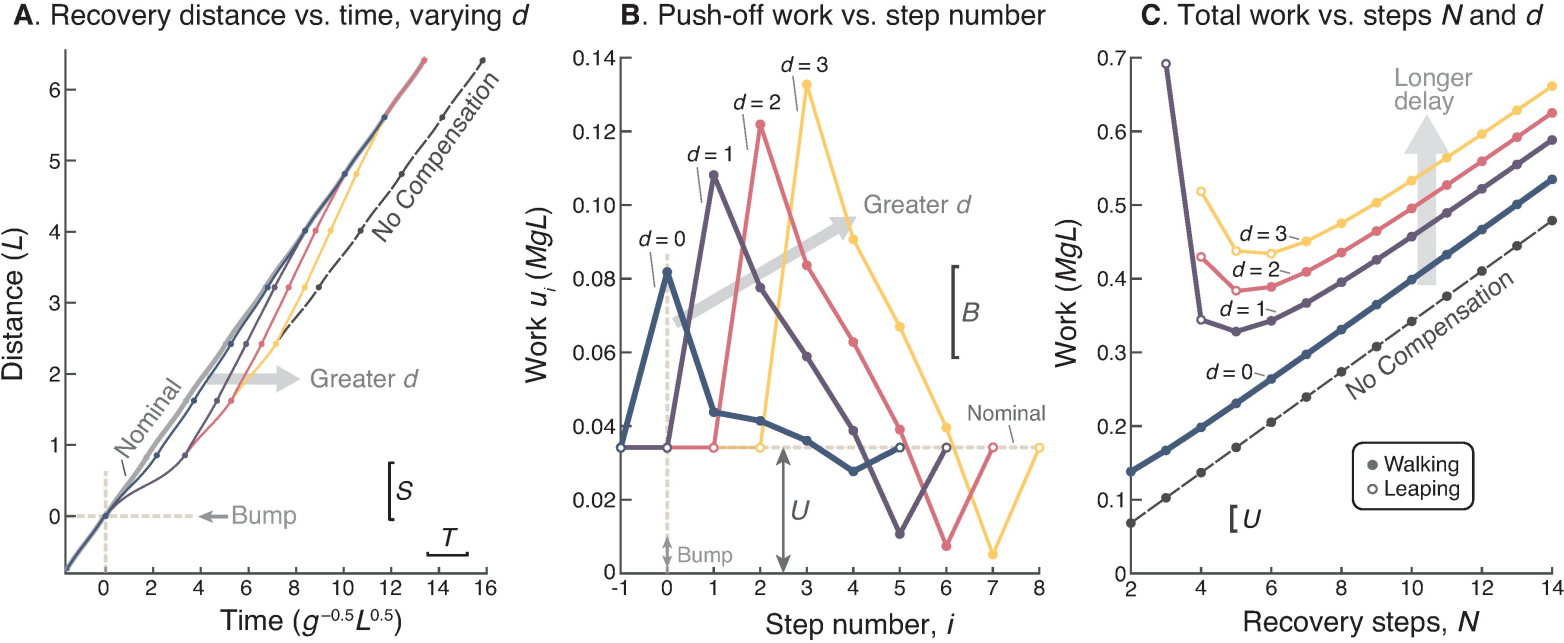
Effect of delay *d* on recovery strategy. (A.) Cumulative distance vs. time for recovery delayed by *d* steps, making up for lost time within *N* = 5 steps. (B.) Optimal push-off sequences *u*_*i*_ vs. step number for varying *d* (bump at step number 0). (C.) Total cumulative positive work vs. *N* for various delay *d*; cumulative refers to the summed work of sequences from (B.), without subtracting nominal work as in Fig 2C. Open symbols denote steps where model tends to leap off the bump.

Delayed onset of compensation results in greater loss of time. Starting with the early collision stepping onto the bump, the walking speed is immediately reduced unless immediate compensation is performed (*d* = 0, Fig 3A). Each additional step taken without adjusting push-off (*d* > 0) only magnifies the time lost (Fig 3A), with the result that the recovery strategy increases in amplitude with greater delay (Fig 3B). Considerably more work must be expended in total for the compensatory recovery (Fig 3C). As an example, for five step recovery (*N* = 5), even a single step delay (*d* = 1) requires twice as much (2.051 times) work than zero delay (*d* = 0). An additional step delay (*d* = 2) requires 2.406 times as much work.

Delaying compensation also makes it more difficult to catch up quickly. For delay (*d* > 0), the amount of push-off needed to catch up in a few (3 to 5) steps calls for the model to leap off the bump (open symbols, Fig 3C), rather than walk. We did not allow the model to benefit from an aerial phase (which confounds the constant step length constraint) when leaping off the bump, and use leaping as an indicator of an especially costly recovery strategy. Overall, it remains highly advantageous to respond without delay, using a late push-off when stepping onto the bump.

### 5. Higher bumps require more work

**Fig 4:**
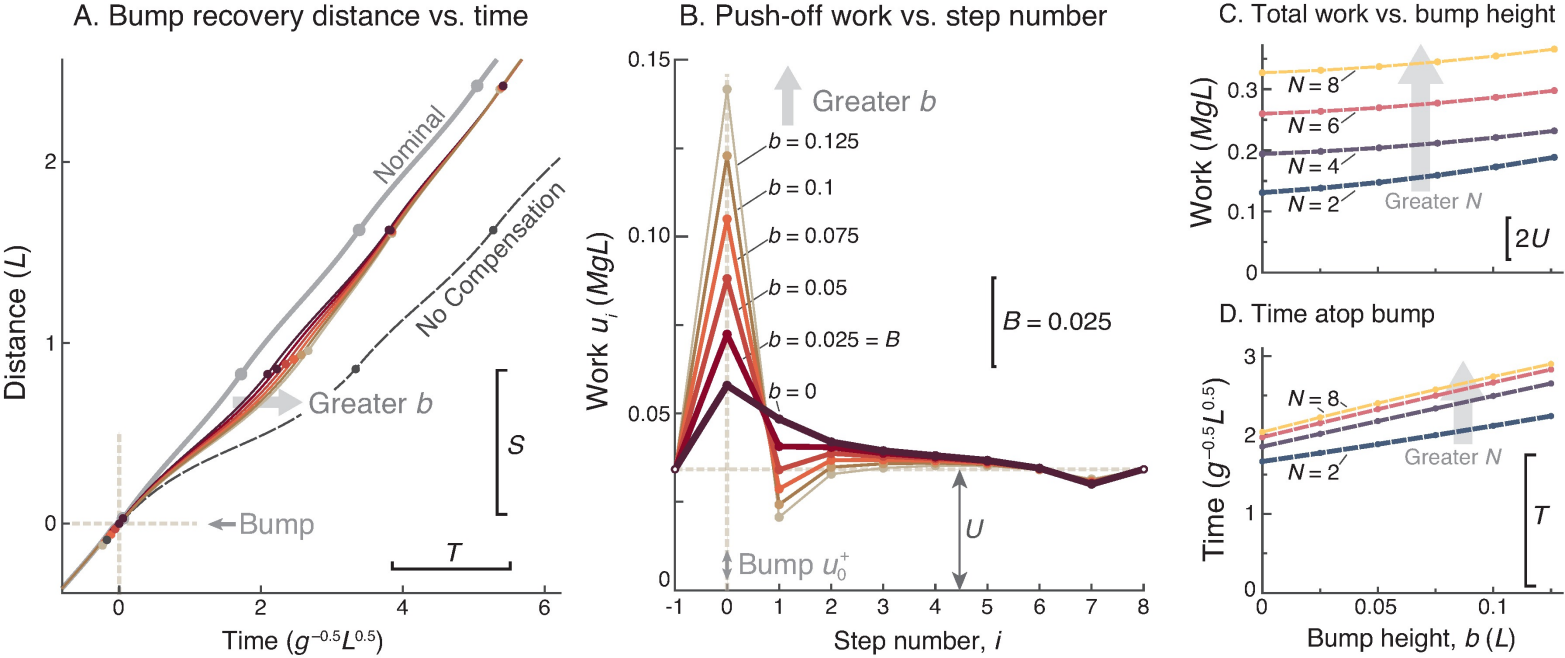
Effect of bump height *b* on recovery strategy. (A.) Cumulative distance vs. time for various bump heights, recovering with *N* ranging 2–8 steps and zero delay (only the first 3 steps shown for legibility). (B.) Optimal push-off sequences *u*_*i*_ vs. step number for varying *b*. (C.) Total positive work as function of *b*, for recovery within *N* = 2 and 8 steps; total work is compared to the positive work to take the same number of nominal, level steps. (D.) Step time (*τ*_0_) atop the bump, as function of bump height *b* for *N* = 2 and 8.

Walking over a higher bump causes a greater loss of time, and a compensatory response of greater amplitude (Fig 4). Bump height *b* determines the amount of time lost atop the bump (Fig 4A), and therefore calls for a recovery strategy of greater amplitude with bump height (Fig 4B). The total work cost of overcoming a bump increases approximately quadratically with bump height (Fig 4C). For example, a bump twice as high as the basic *B* (0.05*L* vs. 0.025*L*), costs 6.8% more to recover from. Associated with this work, the time lost atop the bump (*τ*_0_) increases with bump height (Fig 4D). Another feature of the recovery is that the push-off from atop the bump (*u*_*i*_) is reduced in magnitude with greater bump height. Less push-off is needed because more speed is gained from stepping down a greater distance (Fig 4B).

### 6. Similar rules apply to a pure up step

**Fig 5:**
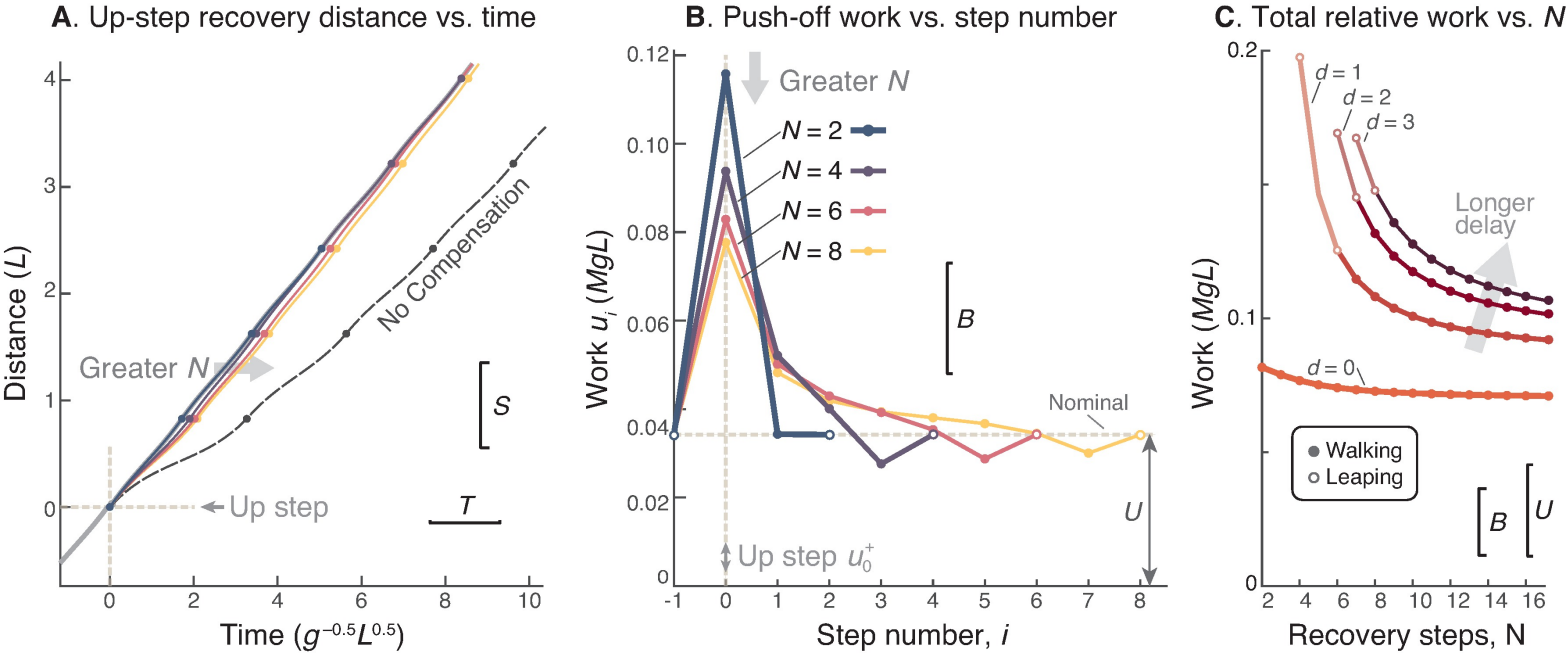
Recovery strategies for a single up step of height *b* (as opposed to up-and-down over a bump). (A.) Cumulative distance vs. time for various number of steps *N*, with zero delay. (B.) Optimal push-off sequences *u*_*i*_ vs. step number for varying *N*. (C.) Total additional positive work to recover as function of *N*, for recovery within *N* ranging 2–16 steps and varying delay *d*. Curves show total work cost over *N* steps, minus nominal work (*N* + *d*).

A variation on the up-and-down motion over a bump is to a single *up step*, to a higher and constant surface height, such as up a sidewalk curb. If the up step is unexpected, then the optimal strategy is remarkably similar to the optimum for the bump (Fig 5). The up step causes a time lag in a plot of cumulative distance vs. time (Fig 5A), and calls for a strong initial push-off $u_{0}^{+}$ followed by weaker subsequent push-offs. Because there is no step down, the second push-off *u*_1_ is of greater work than when stepping off the bump (compare Fig 5B and Fig 2B). As a result, the total additional work for the up step is slightly greater than for the bump (compare Fig 5C and Fig 2C).

### 7. Time may be traded for work

**Fig 6:**
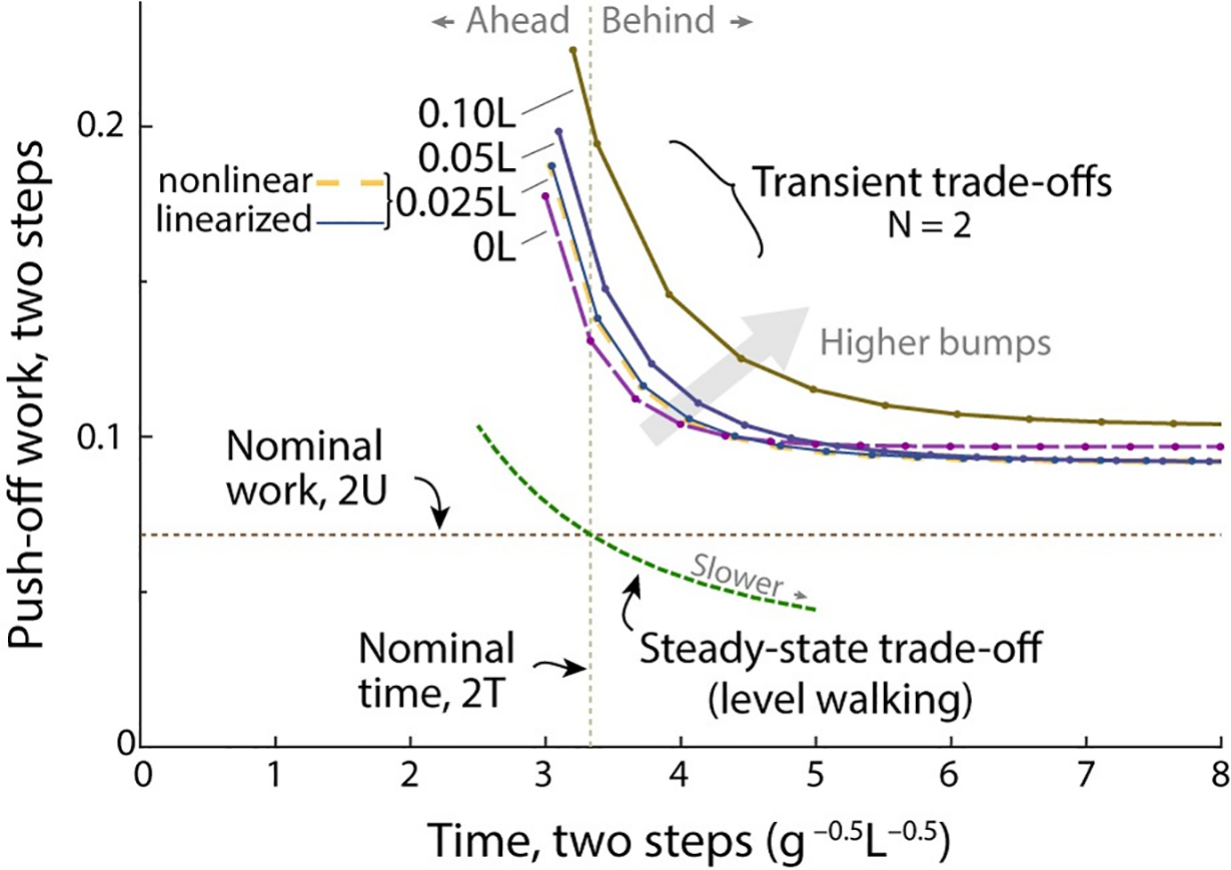
Trade-off between time and work, for two-step recovery from a bump. The nominal gait is represented by the time vs. positive work needed to take two steps of level, steady walking (*Steady-state trade-off*), as a function of varying two-step time (varied above and below the nominal 2*T*, vertical dashed line). These constitute economical options for steady walking at faster or slower speeds. A *Transient trade-off* curve for the standard bump (height 0.025 *L*), showing optimal responses for recovering from a bump in two steps (*N* = 2), taking more or less time than nominal. Various curves show cost for different bump heights. Also shown is the curve for a fully nonlinear model (compare “nonlinear” and “linearized,” both *B* = 0.025 *L*).

There is also a continuum of possibilities for making up more or less time after a bump. If the time constraint is varied above and below nominal, the optimal recovery strategies will vary in work, tracing out a trade-off curve for transient recovery (Fig 6). This curve shows how an allotment of additional time—an amount by which the model will steadily lag behind nominal—allows less work to be expended. As expected, a small increase in allowable time requires much less work. Conversely, if time is to be gained relative to nominal, much more work is needed.

Of course, steady walking also has a trade-off curve (Fig 6), where higher average speed costs more work, which can also be regarded as a time vs. work trade-off. Steady-state walking has a work cost per time that is both lower and less steep than the transient trade-off curve. Unsurprisingly, it is less costly to gain time by selecting a steady average speed over many steps, rather than transiently speeding up for only a few.

Yet another case is when the bump can be anticipated. For example, a single pre-emptive push-off onto the bump could be modulated to restore nominal speed instantly (Eq 3), at considerably lower cost than a late push-off (Eq 9). Indeed, still more energy could potentially saved with earlier anticipation, by modulating additional (pre-emptive) push-offs leading up to the bump, and thus gaining time over more steps and with lower overall cost (e.g. steady-state trade-off curve, Fig 6). Although we do not explore such pre-compensation strategies here, it is clear that an unanticipated bump with late push-off either entails a loss of time relative to nominal or requires considerably more work to regain nominal speed and timing.

## Discussion

The optimization was intended to resolve how best to make up for lost time after an unexpected bump. Considerable time is lost from the bump, and a sequence of once-per-step control inputs was sought to catch up to the nominal, level gait, optimizing for positive work. The optimal sequence was examined in terms of two main free parameters, revealing a high cost to a delay *d* in commencing the recovery strategy, and a much more modest cost for compensating in fewer rather than more steps *N*. This pattern was quite consistent, such that insight may be gained by first focusing on the two-step case before examining on more steps. We therefore begin with that perspective in considering how to recover from a bump in the road.

The main consequence of an unexpected bump is a slow-down, which may be separated into two effects. First and foremost is the energy dissipated by an early collision. An unanticipated bump disrupts the advantage of pushing off pre-emptively, which would reduce the energy lost in collision [15]. Releasing the same amount of energy later, as a mid-stance impulse, is insufficient to regain nominal speed. This slow-down is accompanied by a second and smaller effect, from the exchange of kinetic energy for potential energy at mid-stance. The greater effect of early collision is illustrated by a hypothetical bump of zero height, which still requires considerable work to recover from (Fig 4). In fact, a late push-off with no bump requires more work by itself, than the additional work needed to recover from a bump of height 0.1*L* (compare with 0*L*, Fig 6). Most of the speed and time expense of a bump comes from the lack of pre-emptive push-off.

Regardless of what causes the slow-down, the compensation requires added mechanical work. Time must be regained starting as soon as possible, preferably a strong, if late, push-off ($u_{0}^{+}$). On level ground, late push-off is generally less uneconomical than pre-emptive push-off, but here it is still better than the alternative of waiting for the next push-off opportunity. Waiting would mean so much time lost from the slow stance phase atop the bump (see Fig 3, *d* > 0), that far more work would be needed to speed up with succeeding pre-emptive push-offs. We also found that, after the late push-off, subsequent pre-emptive push-offs are helpful for matching the final condition in speed (*N* ≥ 2; Fig 2B). And the main advantage of taking more steps is a decrease in peak push-off amplitude, thus reducing peak actuation demands. Energetically, there is only modest penalty for recovering in two steps compared to many (Fig 2C).

These conclusions are based on a simple model with discrete ordering of impulsive push-off and collision. Humans actually perform push-off and collision over partially overlapping, finite time [11,16], such that push-off need not completely precede the beginning of collision for good economy. The present model may thus overemphasize the penalty for slightly late push-offs in humans. Certainly, no real-world terrain is perfectly flat, and small surface imperfections do not prevent humans from performing pre-emptive push-off. However, on terrain with about ±2.5 cm variations, humans do perform much less ankle push-off, and rely more on mid-stance powering (similar to late push-off here), with about a 28% penalty in walking economy [17]. This suggests that there is a threshold for bump height that does appear to disrupt pre-emptive push-off in humans.

Our results are also based on a single combination of walking speed and step length. Examining parameter variations, it is evident that the work cost per step increases with the square of speed (Eq 2). Faster walking therefore amplifies overall cost of locomotion, but does not alter the basic strategy for regaining lost time. Similarly, the cost per step also increases approximately with the square of step length (parameterized by *α*). Greater step length causes a faster dissipation of momentum with each step (by a factor cos 2*α* per step, Eq 3). This factor contributes to the decreasing advantage to recovering in many steps (say, *N* > 6). And conversely, taking shorter steps would increase retention of momentum, and make it more viable to recover over more steps. In humans, speed and step length normally increase together [18,19], thus combining these separate effects. The present model should not be regarded as an accurate model of humans, but rather a crude illustration of the basic principles that might govern their recovery strategies.

There are of course other options for recovering from an unexpected bump. One option is to do nothing (No Compensation, Fig 2), and simply apply nominal work each step (including a late impulse atop the bump). This will cause gait speed to asymptotically approach normal, albeit slightly behind (1.51 steps in the example of Fig 2). However, if time permits such a slow-down, then there may have been a slower, more economical nominal gait to begin with (Fig 6). Or perhaps the nominal speed could be selected to select task demands, with some time margin based on a probability of unexpected disturbances. The transient work-time trade-off (Fig 6) could be used to select such a speed.

Another possible strategy might be to allow deviation from the inverted pendulum gait, by bending the stance leg at the knee while going over the bump. This would allow the COM to follow a lower and less arced trajectory, and thus lose less speed. Indeed, the “linear inverted pendulum” model [20] keeps the COM at constant height, and could maintain a level path despite a transient bump. The dynamics of such a gait are quite different, because there are no collisions. However, a bump would still be expected to cause a loss of speed and time, due to an effective loss of COM height above the bump. The trade-offs would differ from those examined here, but we would still expect bumpy terrain to adversely affect walking speed unless compensated for.

The present study did not examine the possibility of modulating swing leg motion. In steady state, taking shorter steps will generally entail reduced collision costs (Fig 4). But these savings are presumably counteracted by greater costs for faster swing leg motion [21,22], thus favoring an optimum step length of intermediate distance [3,19]. Similar trade-offs would be expected to apply to the transient following an unanticipated bump, where lost speed and time are to be regained. Again, the collision costs could be reduced with shorter steps, but at higher cost for shorter step periods. It is possible that greater overall economy could be gained by modulating step lengths after a bump rather than keeping them constant, but the potential savings would depend on the details of a presumed cost for modulating the swing leg.

We also did not consider a torso or other degrees of freedom in our model, which could affect the dynamics substantially if controlled appropriately. For example, collision costs could theoretically be avoided altogether if the torso could be pitched forward and backward by a passive elastic hip spring [23]. However, humans do not obviously adopt large torso accelerations for small bumps. Nor do the robots considered here, which employ physical or virtual constraints [4,5,24] to reduce the effective degrees of freedom and help guarantee stability. But there may be advantage to intentionally moving or even flailing body parts in response to extreme disturbances. We would expect such motions to have relatively low control authority for balance, compared to the fundamental collision effect modeled here [25]. Even with more degrees of freedom, a robot or human with pendulum-like dynamics would still be expected to experience a qualitatively similar collision with a bump (e.g., conserving angular momentum about the contact point), and experience a qualitatively similar loss of speed and time.

This study also regarded regulation of speed as separate from stabilization. We treated speed regulation as a higher-level, multi-step issue, and stability as a lower-level, single step issue. By constraining step lengths or employing virtual holonomic constraintddecas, stability is assured, leaving speed regulation as the remaining issue. Although these constraints simplify control, they may also have disadvantages. For example, pushing off harder to maintain speed might demand greater control effort to satisfy virtual constraints (e.g., to keep the trunk upright). There might well be alternative control strategies that are less constrained but more robust [26–29]. In addition, frontal plane stability also requires control, such as with lateral foot placement [25,30]. It may be advantageous to pose a single optimization problem to integrate stability in three-dimensions with regulation of forward speed.

This is a relatively simple study with a number of limitations. More detailed modeling would be necessary to capture details of human or robot mass distribution and other degrees of freedom such as knees [31], ankles [14,32], or a torso. Other features could include the human speed-step length relationship [19], ground clearance during swing [33], and different ways the foot might contact a bump [34]. These features could potentially modify the basic strategy for recovering from a bump.

This study also focused only on unanticipated bumps that affect pre-emptive push-off. We would expect that the ability to anticipate a bump, and either adjust push-off timing or plan other strategies, might be preferable to the recovery strategies examined here. However, it is also reasonable to assume that some bumps will be unseen and necessitate a post-hoc recovery. Finally, we treated time and work as the main variables of interest. More realistic costs might include explicit actuator limitations, actual energy cost, and a host of other variables related to task demands.

This study provides a quantitative counterpart to the old adage that time is money (work). Assuming that walking means to achieve some goal, there may be a disadvantage to arriving late, and an unexpected bump in the road increases lateness, and might thus affect the achievement. Fortunately, there are relatively simple recovery strategies to make up for lost speed and time, in as few as two steps. Such strategies could potentially be triggered automatically after the bump, thus reducing the need to reevaluate an entire task optimization because of such an unforeseen circumstance. By translating the recovery into a trade-off between time and work, it is also possible to evaluate other options, such as whether it might actually be worth it to arrive late, due to the cost of making up time. Locomotion costs both time and energy, and both costs should contribute to task planning and decision making.

### List of mathematical symbols

*θ* Stance leg angle, positive clockwise from vertical
*α* Half-angle between the legs
*b*,*B* Variable bump height, and standard bump height for comparison (units *L*)
*S* Fixed step length, *S* = 2 sin *α* (units *L*)
*τ*_*i*_ Stance phase duration (step time) for step *i* (units *g*^−0.5^*L*^0.5^)
*u*_*i*_ Push-off work for step *i*, normalized by mass *M* (units *gL*)
$u_{0}^{+}$ Mid-stance impulsive work for step 0, normalized by mass *M* (units *gL*)
*T*,*U* Nominal step time and push-off for level, steady walking
$v_{i}^{-}$ COM velocity magnitude immediately before push-off, step *i* (in units *g*^0.5^*L*^0.5^)
$v_{i}^{0}$ COM velocity magnitude immediately after push-off and before collision, step *i*
$v_{i}^{+}$ COM velocity magnitude immediately after collision and before early-stance acceleration, step *i*
$v_{0}^{0}$ COM velocity immediately after collision and mid-stance input $u_{0}^{+}$, step *i*
*V* Nominal COM velocity before or after step-to-step transition for level, steady walking
*M*,*L*,*g* Body mass, leg length, gravitational acceleration as base units for non-dimensionalization
